# Effects of monochromatic infrared energy in patients with diabetic peripheral neuropathy: a meta-analysis of randomized clinical trials

**DOI:** 10.1186/1758-5996-7-S1-A16

**Published:** 2015-11-11

**Authors:** Caroline Cabral Robinson, Patrícia S Klahr, Cinara Stein, Graciele Sbruzzi, Rodrigo Della Méa Plentz

**Affiliations:** 1 Universidade Federal de Ciências da Saúde de Porto Alegre Porto Alegre, Brazil

## Background

Monochromatic infrared energy (MIRE), delivered through light-emitting diodes, has been used as a non-pharmacological complementary strategy to improve plantar sensitivity and pain symptoms in patients with diabetic peripheral neuropathy (DPN), but conflicting results[1,2] have been reported.

## Objective

Summarize the effect of MIRE in plantar sensitivity and neuropathic pain in patients with DPN trough a systematic review of randomized clinical trials.

## Materials and methods

MEDLINE, EMBASE, Cochrane Central and Google Scholar were searched for studies published up to May 2015. Two independent reviewers assessed study eligibility based on predefined criteria and performed data extraction. Results of plantar sensitivity were in standard mean difference, and pain were in mean difference, with 95% of confidence intervals. Statistical heterogeneity was assessed by Cochran's Q test and inconsistency I2 test. A p value ≤ 0.05 was statistically significant. Meta-analysis was performed on RevMan 5.3.

## Results

**Figure 1:**
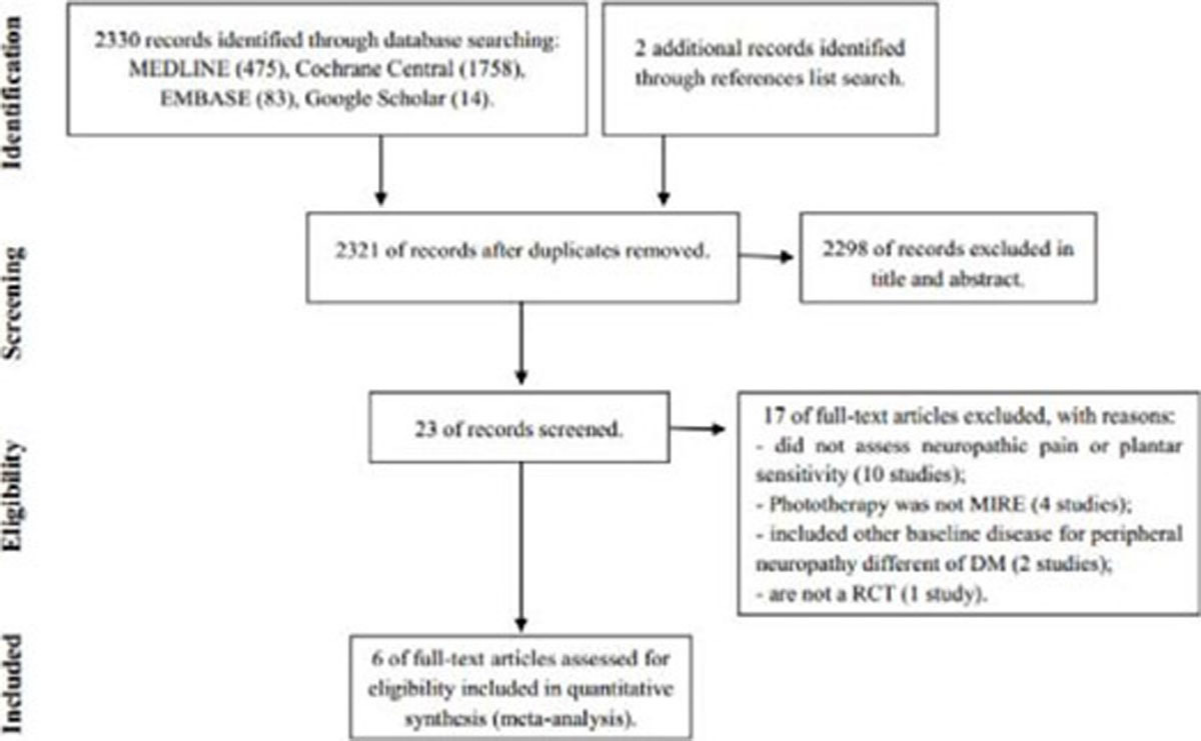
Flow diagram of studies included.

**Figure 2:**
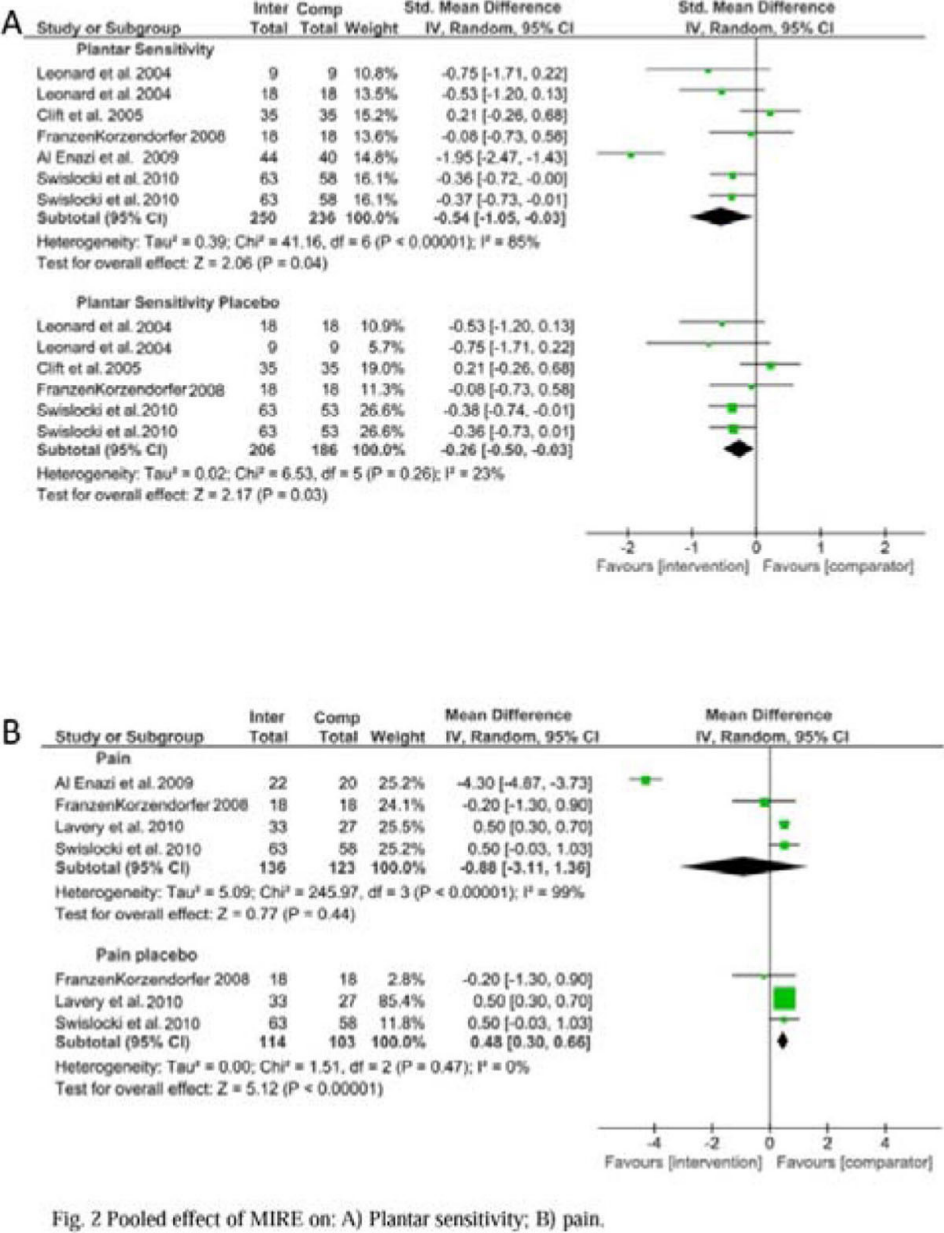
Effect of MIRE on: A) Plantar sensitivity; B) Pain.

From 2330 abstracts, six studies met the eligibility criteria and were included in the systematic review (304 patients; 606 feet) (Figure 1). Participants were adult individuals with type 1 or 2 diabetes and DPN. MIRE was applied for at least thrice a week for 30 min/day in ankles and plantar aspect of feet. Follow-up ranged from two to 12 weeks. Comparison group (placebo or control) did not receive MIRE. Overall effect of MIRE in plantar sensitivity was a statistically significant reduction in insensitive plantar areas to the 5.07 Semmes-Weinstein monofilament [–0.54(–1.05 to –0.03); I2: 85%]. Heterogeneity decreased after a sensitivity analysis including only placebo studies; effect size remained statistically significant favoring MIRE [–0.26(–0.50 to–0.03); I2: 23%]. Overall pain symptoms decreased but not differed between MIRE and comparison groups [–0.88(–3.11 to 1.36); I2: 99%]. After a sensitivity analysis including only placebo studies, heterogeneity decreased but a statistically significant placebo effect was found in pain relief [0.48(0.30 to 0.66); I2: 0%] (Figure 2).

## Conclusion

MIRE slightly improves plantar sensitivity in DPN with moderate confidence; further well-designed studies were likely to change effect size and reduce heterogeneity.
